# Safety and efficacy of catheter ablation in atrial fibrillation patients with left ventricular dysfunction

**DOI:** 10.1002/clc.23314

**Published:** 2019-12-05

**Authors:** Songbing Long, Yutao Xi, Lianjun Gao, Qi Chen, Jie Cheng, Yanzong Yang, Yunlong Xia, Xiaomeng Yin

**Affiliations:** ^1^ Department of Cardiovascular, The Central Hospital of Shaoyang Shaoyang China; ^2^ Department of Cardiovascular, Texas Heart Institute Houston Texas; ^3^ Department of Cardiovascular, The First Affiliated Hospital of Dalian Medical University Dalian China

**Keywords:** antiarrhythmic drugs, atrial fibrillation, catheter ablation, heart failure, medication therapy

## Abstract

**Background:**

Catheter ablation (CA) for atrial fibrillation (AF) in heart failure (HF) patients reduced the mortality but may increase complications and raise the safety concern.

**Hypothesis:**

CA for AF in HF patients may not increase the complications vs medical treatment, and it may reduce hospitalizations and mortality and improve heart function.

**Methods:**

Three groups of AF patients were included in the study: 120 congestive HF for their first CA (AFHF‐CA), 150 congestive HF who were undergoing medical therapy (AFHF‐Med), and 150 patients with normal left ventricular (LV) ejection fraction (LVEF) (AF‐CA).

**Results:**

After 30 ± 6 months of follow up, 45.8% of patients in the AFHF‐CA and 61.3% of patients in the AF‐CA groups maintained sinus rhythm (SR) in comparison with 2.7% in AFHF‐Med (*P* < .01). Hospitalization for HF was significantly lower in AFHF‐CA than in AFHF‐Med groups (*P* < .01). Death occurred in 7.5% of patients in the AFHF‐CA group, which was lower than 18% in the AFHF‐Med group (*P* < .01). Significant improvements in heart function were shown in the AFHF‐CA group compared to the AFHF‐Med group, including LVEF (*P* < .01), LV end‐diastolic diameter (*P* < .01), and New York Heart Association classification (*P* < .01), as well as the left atrial diameter (*P* < .01). AFHF‐CA patients required additional ablation more often (*P* < .05). CA had a better prognosis in paroxysmal AF and tachycardia‐related diseases.

**Conclusion:**

CA for AF reduced hospitalizations and mortality and improved heart function, vs medical treatment, and was as safe as CA in those with normal heart function.

## 1 INTRODUCTION

Atrial fibrillation (AF) and congestive heart failure (HF) are the most common heart diseases and frequently coexist.^1^ HF predisposes to AF with a prevalence of around 30%.^2^ AF can aggravate HF and increase mortality over 2‐fold.^3^, ^4^, ^5^, ^6^ Successful restoration and maintaining of sinus rhythm (SR) by catheter ablation (CA) significantly improved the cardiac function and outcome of HF compared to the antiarrhythmic drug.^7^, ^8^ However, the ambivalence increased complications in HF patients and may raise safety issues.

Nevertheless, few random control trials and numerous retrospective observational studies with fewer than 100 subjects indicated that ablation in a patient with HF has similar overall efficacy rates as those without HF. However, recently, the AATAC trial (Ablation Versus Amiodarone for Treatment of Persistent Atrial Fibrillation in Patients With Congestive Heart Failure and an Implanted Device),^9^ and CAMERA‐MRI trial (Catheter Ablation Versus Medical Rate control in Atrial Fibrillation and Systolic Dysfunction)^10^ indicated that CA in HF patients improved^11^ freedom from AF and reduced mortality and hospitalization rates, compared to amiodarone therapy. Moreover, the most recent study, CASTLE AF (Catheter Ablation Versus Standard Conventional Treatment in Patients with Left Ventricular Dysfunction and Atrial Fibrillation),^11^ just reported that the all‐cause mortality and hospitalization for HF were significantly lower in the CA group. However, antiarrhythmic drugs, such as amiodarone (over 85%), were used as the primary therapy in these studies, and burden of AF and no data on concurrent cardiac heart diseases may limit the application of results in practice.^12^, ^13^ Currently, the RAFT AF trial (A Randomized Ablation‐based Atrial Fibrillation Rhythm Control Trial in Patients with Heart Failure and High Burden Atrial Fibrillation, NCT01420393, http://clinicaltrials.gov) is ongoing to evaluate the CA benefits in a subgroup of patients.

However, more evidence is needed to support CA as first‐line therapy for precision AF management in patients with HF regarding the long‐term outcome and prognosis.^14^, ^15^

The present study was sought to investigate the long‐term efficacy of CA in patients with HF by comparing pharmacological approaches and the safety of CA by comparing AF patients with or without HF. Most importantly, subgroups analysis on the etiology of HF and burdens of AF were investigated to provide evidence in patient selection and personalized AF therapies.

## 2 METHODS

### 2.1 Patient population

Patients who underwent treatment for symptoms of AF at the First Affiliated Hospital of Dalian Medical University between May 2006 and April 2013, according to clinical diagnosis of AF and HF, were included in the study. HF was defined as left ventricular (LV) ejection fraction (LVEF) ≤ 50% and New York Heart Association (NYHA) class ≥ II.^16^ The definition and classification of AF were based on published guidelines from the American College of Cardiology‐American Heart Association and the European Society of Cardiology.^17^

Inclusion criteria were first‐time CA of symptomatic, drug‐refractory AF and no detected intracardiac thrombus. Exclusion criteria was postoperative AF, with a pacemaker or implantable cardioverter defibrillator; history of stroke; and severe pulmonary, renal, or liver diseases. The study protocol was preapproved by the university's Research Development and Human Ethics Committee. Before enrolling in the study, all patients signed the written informed consent. All antiarrhythmic medications were suspended for at least five half‐lives before the procedure. Three groups of AF patients who underwent treatment were enrolled: 120 patients with congestive HF (AFHF‐CA); 150 patients with congestive HF who were undergoing medical therapies (AFHF‐Med); and 150 patients with normal heart function (AF‐CA) who have matched age, gender, and type of AF with AFHF‐CA patients.

### 2.2 Baseline evaluation

In all patients, transthoracic echocardiography was performed at baseline (2 ± 1 days before ablation) to evaluate the size of the left atrium and LV function. Transesophageal echocardiography was performed before CA of AF to assess LV systolic function and to rule out intracardiac thrombus. A 24‐hour ambulatory electrocardiography was performed before CA of AF (ie, 1‐2 days) to monitor heart rate and heart rhythm.

### 2.3 Electrophysiological study and radiofrequency CA

Oral anticoagulation (OAC) therapy with phenprocoumon was stopped at least 1 day before ablation and bridged with low‐molecular‐weight heparin. In all cases, lidocaine was administered for local anesthesia at the catheter access site. Patients were heparinized to activate clotting time >250 seconds, right after double transseptal puncture access using a modified Brockenbrough technique. Circumferential pulmonary vein (PV) isolation (CPVI) is the initial ablation approach used in all AF patients who underwent ablation, followed by additional substrate modification, including left atrial linear ablation and isthmus ablation. The following catheters were inserted in the femoral vein and positioned: (a) distal 10 poles (Webster Fixed Curve Catheter; Biosense Webster, Diamond Bar, California) at coronary sinus; (b) distal four poles (Webster Fixed Curve Catheter; Biosense Webster) at right ventricle; (c) a spiral decapolar mapping catheter (Lasso; Biosense Webster) at the target PV through SL1 transseptal sheath (Intracardiac Catheter Introducer Kit and Transseptal Needle, Synaptic Medical, Beijing, China); and (4) a conventional 3.5 mm irrigated‐tip ablation catheter (ThermoCool Navi‐Star; Biosense Webster) combination with CARTO mapping system (Biosense Webster) to reconstruct a three‐dimensional electroanatomical LA through SL1 transseptal sheath. The selective angiography of each PV using left anterior oblique (LAO) 45° and LAO 40° fluoroscopic views was performed.

### 2.4 CA protocol

All patients underwent CPVI using irrigated RF with a maximum temperature of 43°C, a maximal power of 40 W, and an infusion rate of 17 to 25 mL/min, and power was limited to 30 W at the posterior wall. CPVI was performed with a spiral mapping catheter. The endpoint of CPVI was defined as the absence of any PV spike potential in the spiral‐mapping catheter inside the lateral PVs. Ablation of complex fractionated atrial electrograms (CFAE) guided by an LA CFAE map was performed if SR could not be achieved after CPVI. If frequent atrial premature beats or atrial tachycardia (AT) occurred, superior vena cava (SVC) isolation was performed. A cavotricuspid isthmus (CTI) ablation was performed if familiar atrial flutter occurred. If AF persisted, SR was restored by cardioversion with two biphasic direct current shocks (150 and 200 J). For patients with recurrent AF or AT, a further ablation was performed to isolate the PV conduction gaps completely, and furthermore, SVC isolation, CTI ablation, and focal ablation for AT were also performed to achieve a bidirectional block. The maximal temperature of ablation was 43°C, with a power range of 30 to 40 W and an irrigation speed of 17 to 25 mL/min. In particular, for the posterior atrial wall, the power was set to no more than 30 W.

### 2.5 Medical therapy strategies

Patients in medical therapy (AFHF‐Med) received amiodarone, propafenone, beta‐blockers, or digoxin and combinations during the predefined 3‐month blanking period targeted to achieve a mean heart rate (assessed by apical auscultation over 30 seconds) of 90 beats/min at rest before and <110 beats/min after a 6‐minute walk.^8^, ^18^ The therapies were continued throughout the follow‐up periods and were adjusted to meet the goal of rate control. OAC therapy was prescribed for all patients in AFHF‐Med group. Patients in the AFHF‐CA and AF‐CA groups received OAC therapy 2 months after the procedure. This would be extended to 6 months when the CHA_2_DS_2_‐VASc ≥ 2; then, OAC therapy was stopped if there was no more recurrence.

### 2.6 Postablation follow up

Anticoagulant therapy with warfarin targeted an international normalized ratio of 2 to 3 postprocedure and was continued for a minimum of 3 months after OAC therapy was prescribed based on the patient's CHA_2_DS_2_‐VASc score.^19^ One day after ablation, a 24‐hour Holter recording was used to evaluate the heart rhythm and heart rate, and a transthoracic echocardiogram (TTE) was performed to exclude pericardial effusion. Medicine therapy was recommended for 3 months after the procedures in patients who remained in SR. All patients were scheduled for visits in the outpatient clinic every month for the first 3 months after the procedure. The recurrence of AF was evaluated by symptoms, 12‐lead electrocardiogram, 24‐hour ambulatory monitoring (3, 6, 9, and 12 months and every 6 months after that). A TTE was performed in all patients at the same time points to evaluate the LV function. Recurrence of AF/AT was defined as recurrent symptoms and documented AT lasting >30 seconds on the electrocardiogram or 24‐hour ambulatory monitoring after a 3‐month blanking period from the ablation procedure.

### 2.7 Statistical analysis

Categorical data were described as counts and percentage, and continuous data were expressed as a mean ± SD. A comparison between two groups was performed with Student's *t* test. A comparison between the three groups was performed with analysis of variance if appropriate and the Kruskal‐Wallis test otherwise. Changes between two time points (baseline and the follow ups) were analyzed with paired *t* tests or with the Wilcoxon signed rank test on continuous variables, and categorical variables were evaluated with the chi‐square test or with Fisher's exact test. The survival rate was estimated by the Kaplan‐Meier method and compared by the log‐rank test, or the Wilcoxon test was applied. Univariate and multivariate analyses with logistic regression were used to evaluate variables for predicting procedural success. The results were reported as odds ratio (OR) with 95% confidence intervals (CIs). All tests of significance were two‐tailed, and a *P* value of <.05 was considered to indicate statistical significance. All analyses were performed by using Stata 13.0 (StataCorp, College Station, Texas) and GraphPad Prism version 5.0 (GraphPad Software Inc., La Jolla, California).

## 3 RESULTS

### 3.1 Basic characteristics of patients

The basic characteristics of the patient population were summarized in Table 1. Recruited patients were an average age of 61 ± 8.7 years. Of the patients, 60% were male and equally distributed in three groups (*P* = 1.00). All types of AF were evenly distributed within three groups (*P* = .57), including 206 (49%) persistent AF patients, 70 (17%) permanent AF patients, and 144 (34%) paroxysmal AF patients, with average an AF duration of 80 ± 83 days (*P* =.843). Attempting SR with electrical cardioversion (*P* = .51) and/or AAD (antiarrhythmic drug) (*P* = .23) was comparable among the three groups. There was a total of 93 (22%) dilated cardiomyopathy (DCM) patients in three groups (*P* < .01). Fewer DCM patients were in AF‐CA group compared to HF groups (*P* < .01). Other concurrent heart diseases, including coronary artery heart diseases (62, 14.8%), valvular diseases (56, 13.3%), congenital heart diseases (11, 2.6%), and hypertrophic cardiomyopathy (14, 3.3%), were comparable among three groups. AFHF‐CA and AFHF‐Med have a comparable NYHA class (*P* = .06), LVEF (*P* = .06), LV end‐diastolic diameter (*P* = .12), and left atrial dimension (LAD, *P* = .36). In the AFHF‐Med group, rate control criteria were met in 82 of 150 (54.7%) patients, the beta‐blockers dose was increased in 42, digoxin was added in 20, and digoxin dose was increased in 6 patients.

**Table 1:** Baseline characteristics of patients

	AF‐CA (150)	AFHF‐CA (120)	AFHF‐Med (150)	*P* value of three groups	*P* value of AFHF‐CA vs Med
Clinical characteristics					
Age (y)	59.7 ± 8.3	60.7 ± 9.1	63.1 ± 8.5	.24	.45
Male, *n* (%)	90 (60)	72 (60)	90 (60)	1.00	1.00
Classification of AF					
Paroxysmal, N (%)	48 (32.0)	38 (31.7)	48 (32.0)	1.00	1.00
Persistent, N (%)	74 (49.3)	60 (50.0)	72 (48)	.94	.94
Permanent, N (%)	28 (18.6)	22 (18.3)	20 (13)	1.00	1.00
Duration of AF (mo)	78 ± 68	84 ± 88	80 ± 86	.84	.84
Electrical cardioversion, times (%)	51 (34.0)	36 (30.0)	55 (36.6)	.52	.52
No. of antiarrhythmic drugs tried, N (%)	112 (74.6)	92 (76.6)	102 (68)	.23	.23
Treatment with amiodarone, N (%)	15 (10)	9 (7.5)	12 (8)	.73	.73
Hypertension, N (%)	86 (57.3)	72 (60)	95 (63.3)	.57	.57
Diabetes mellitus, N (%)	23 (15.3)	19 (15.8)	25 (16.7)	.95	.95
CHA_2_DS_2_‐VASc score	1.7 ± 1.1	2.6 ± 1.1	2.8 ± 1.1**	.06	.06
Concurrent heart disease					
DCM alone, N (%)	21 (14)	29 (24.2)	33 (22)**	<.01	.17
Coronary artery disease, N (%)	20 (13.3)	16 (13.3)	26 (17.3)	.54	.54
Valvular disease, N (%)	19 (12.7)	15 (12.5)	22 (14.7)	.83	.83
Congenital heart disease, N (%)	4 (2.7)	2 (1.7)	5 (3.3)	.7	.7
Hypertrophic cardiomyopathy, N (%)	4 (2.7)	3 (2.5)	6 (4)	.65	.65
Heart function					
NYHA functional class	0.3 ± 0.5	2.7 ± 0.6**	2.9 ± 0.6**	<.01	.06
LV ejection fraction (%)	59.1 ± 2.6	41.9 ± 4.6**	40.8 ± 4.8**	<.01	.06
End‐diastolic LV dimensions (mm)	53.6 ± 4.2	60.9 ± 4.3**	61.8 ± 4.8**	<.01	.06
Left atrial parasternal dimension (mm)	40.8 ± 4.6	42.9 ± 4.2	43.6 ± 7.0*	<.01	.36
Concurrent heart disease					
DCM alone, N (%)	21 (14)	29 (24.2)	33 (22)**	<.01	.17
Coronary artery disease, N (%)	20 (13.3)	16 (13.3)	26 (17.3)	.54	.54
Valvular disease, N (%)	19 (12.7)	15 (12.5)	22 (14.7)	.83	.83
Congenital heart disease, N (%)	4 (2.7)	2 (1.7)	5 (3.3)	.7	.7

Abbreviations: AF, atrial fibrillation; CA, catheter ablation; CHA_2_DS_2_‐VASc, congestive heart failure, hypertension, age ≥75 (doubled), diabetes, stroke (doubled), vascular disease, age 65–74, and sex category (female); DCM, dilated cardiomyopathy; HF, heart failure; LV, left ventricular; NYHA, New York Heart Association.

* *P* < .01.

** *P* < .05 vs AF‐CA; ^***^ *P* < .05 vs AFHF‐CA.

### 3.2 Maintenance of SR

No patient was reported as lost to follow up during the period of 30 ± 6 months (range: 27‐35 months). Second ablations were conducted in 11 patients (9.1%) with AFHF‐CA and 15 patients (10.0%) with AF‐CA. The remaining patients refused to undergo a second procedure due to variant reasons. Because repeated deficient procedures were conducted, only results from the first procedure were considered in the present study. At the end of follow up, the AFHF‐CA group had a lower percentage of patients remaining in SR than the AF‐CA group (56/120 [47%] vs 92/150 [61%], *P* < .05). There was no difference in the percentage of patients remaining in SR between patients in the AFHF‐CA and AF‐CA groups at the 12‐month follow up (79/120 [65.8%] vs 107/150 [71%], *P* = .27) and at the 24‐month follow up (68/120 [56.7%] vs 98/150 [65.3%], *P* = .15) (Table S1). In contrast, at the end of follow up, there were only four (2.7%) patients maintaining SR in the AFHF‐Med group, which was significantly lower than the AFHF‐CA group (*P* < .001). Only 25 (17%) patients in the AFHF‐Med group had successful SR maintenance in the 12‐month follow‐up, which was significantly lower than those in the AFHF‐CA group (*P* < .001). The SR maintenance rate continuously decreased to 16 (11%) (vs AFHF‐CA, *P* < .001).

Survival analysis using the Kaplan‐Meier test estimated the maintenance of SR in three groups, as shown in Figure S1. The AFHF‐Med group had the lowest SR maintenance among the three groups (*P* < .01). However, there were no significant differences between the AFHF‐CA and AF‐CA groups during the follow‐up periods (*P* = .07).

### 3.3 HF hospitalizations, stroke, and death

**Figure 1:**
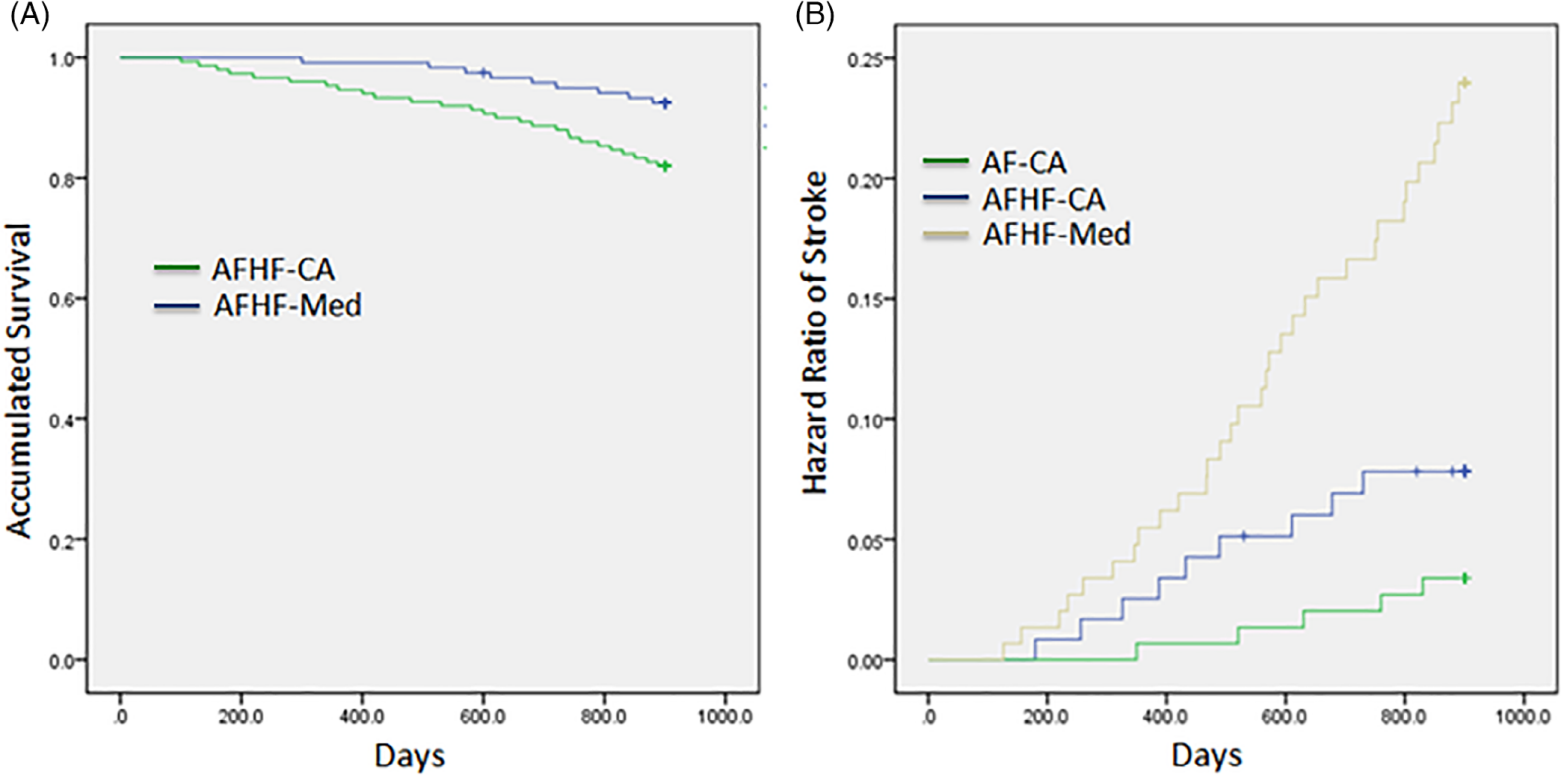
Accumulated survival rate between AFHF‐CA group and AFHF‐Med group, B, the hazard ratio of stroke among the three groups

Over 30‐month follow up, HF hospitalization was significantly lower in AFHF‐CA (1.8 ± 1.4 times) than in the AFHF‐Med groups (3.1 ± 1.0 times, *P* < .01). Stroke occurred in 11 of 120 (9.2%) patients in the AFHF‐CA group during the follow‐up period, which was substantially lower than those in the AFHF‐Med group, about 32 of 150 (21.3%) (*P* < .01), but was significantly higher than those in the AF‐CA group, as shown in Figure 1B by the Kaplan‐Meier graph. Death occurred in 9 of 120 (7.5%) patients in the AFHF‐CA group, in which the cause of death was 6 cardiac, 1 noncardiac, and 2 unknowns. However, in the AFHF‐Med group, death occurred in 27 of 150 (18%) patients (*P* < .01 vs AFHF‐CA), in which the cause of death was 17 cardiac, 3 noncardiac, and 7 unknowns. The Kaplan‐Meier analysis showed significantly different survival rates between the AFHF‐CA and AFHF‐Med groups (Figure 1A). There was no case reported for HF hospitalization, stroke, and death in the AF‐CA group during the observation period.

### 3.4 Improvements in heart function

**Figure 2:**
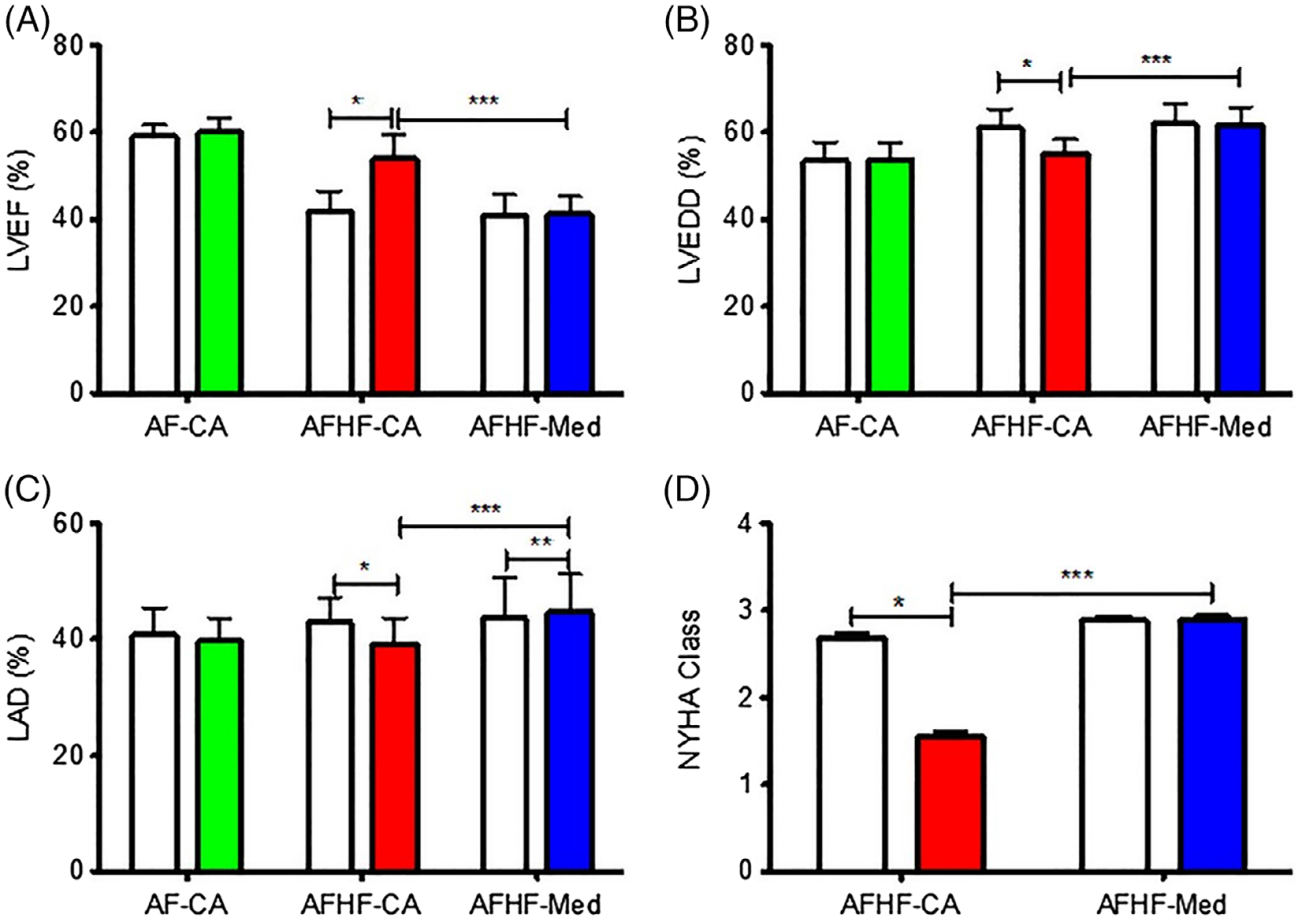
Improvements of heart function between baseline and after 30‐month follow up. A, Left ventricular ejection fraction. B, Left ventricular end of diastolic dimension. C, Left atrial dimension. D, New York Heart Function Classification. *P < .01 (AFHF‐CA group). **P < .01 vs AF‐CA (AFHF‐Med group); ***P < .001 (AFHF‐CA group VS AFHF‐Med group)

After 30‐month follow up, changes are shown in Figure 2. Compared to the AFHA‐CA group, patients in the AFHF‐Med group had worse LVEF (*P* < .001), larger LV end of diastolic dimension (LVEDd, *P* < .001), and LAD (*P* < .001), and higher NYHA class (*P* < .001).

Significant improvements were only detected in the AFHF‐CA group at the end of the follow up. Using pair tests between baseline and after 30‐month follow up, significant improvement in LVEF was detected in AFHF‐CA group from 41.9 ± 0.43% to 53.8 ± 0.54% (*P* < .01) (Figure 2A) but not in the AFHF‐Med group (from 40.79 ± 0.43% to 41.1 ± 0.37%, *P* = .89), and median LVEDd decreased in the AFHF‐CA group from 60.9 ± 0.41 to 55.2 ± 0.30 mm (*P* < .01) but not in the AFHF‐Med group (61.8 ± 0.42 vs 61.7 ± 0.35 mm, *P* = .66) (Figure 2B). Furthermore, median LAD decreased in the AFHF‐CA group from 42.9 ± 0.40 to 39.2 ± 0.42 mm (*P* < .01), but LAD was larger in the AFHF‐Med group (43.6 ± 0.64 vs 44.7 ± 0.58 mm, *P* < .01) (Figure 2C). The NYHA classification had a significant improvement in the AFHF‐CA group from 2.68 ± 0.05 at baseline to 1.55 ± 0.06 at 30‐month follow up (*P* < .01) but had no significant change in the AFHF‐Med group (2.88 ± 0.05 vs 2.89 ± 0.05, *P* = .48) (Figure 2D).

### 3.5 CA safety and complications in AF patient with or without HF

Procedural details and complications of CA were summarized in Table S2. PV isolation was successfully performed in all patients. More addition of extralinear ablation was needed in AFHF‐CA patients (86%) than AF‐CA patients (71%) (*P* < .01). Consequently, more ablation time was needed in AFHF‐CA (*P* < .05) than AF‐CA patients. However, the overall procedure time and fluoroscopy time were comparable between the AFHF‐CA and AF‐CA groups. In the AFHF‐CA group, cardiac tamponade occurred in four (3.3%) patients, but two (1.7%) patients did not require percutaneous drainage. Similarly, there were four (2.7%) patients from the AF‐CA group, but two (1.3%) patients did not require percutaneous drainage, which was not a significant difference from the AFHF‐CA group. No stroke occurred during the procedure in all patients.

### 3.6 Benefit of CA in etiology of HF

Three subgroups were analyzed in Table 2 according to major etiology in the AFHF‐CA group patients: 28 DCM; 34 patients with structural heart disease (SHD) other than isolated DCM; and 57 tachycardia‐related cardiomyopathy (sTM), patients without diagnosed SHD, who were therefore suspected to suffer from tachycardia‐induced cardiomyopathy. Compared to baseline, the LVEF had a significant increase in all three subgroups, DCM (53.1 ± 5.5% vs 42.7 ± 3.8%, *P* < .01), SHD (50.2 ± 5.5% vs 40.8 ± 5.0%, *P* < .01), and sTM (56.0 ± 4.0% vs 42.2 ± 4.5%, *P* < .01), after 30‐month follow‐up. The NYHA class significantly improved in DCM (1.71 ± 0.66 vs 2.57 ± 0.57, *P* < .01), SHD (1.61 ± 0.65 vs 2.79 ± 0.64, *P* < .01), and sTM (1.35 ± 0.52 vs 2.63 ± 0.56, *P* < .01).

**Table 2:** Characteristics of patients with DCM, SHD, or sTM

	DCM (28)	SHD (34)	sTM (57)	*P* value
Basic characteristics				
Age (y)	61.4 ± 8.1	64.3 ± 7.9	58.3 ± 9.7	.05
Male (%)	17 (58.6)	16 (45.7)	32 (56.1)	.5
Classification of AF				
Paroxysmal, N (%)	9 (32.1)	7 (20.6)	18 (31.6)	.48
Persistent, N (%)	12 (42.9)	22 (64.87)	25 (43.9)	.11
Permanent, N (%)	8 (28.6)	6 (17.6)	12 (21.1)	.57
Duration of AF (mo)	83 (10 300)	110 (10 400)	68 (10 200)	.27
Electrical cardioversion, times (%)	10 (35.7)	13 (38.2)	16 (28.1)	.57
Antiarrhythmic drugs tried, times (%)	24 (85.7)	28 (82.4)	42 (73.7)	.38
Treatment with amiodarone, no. (%)	3 (10.7)	1 ( 2.9)	5 (8.8)	.46
Hypertension, N (%)	17 (60.7)	22 (64.7)	30 (52.7)	.5
Diabetes mellitus, N (%)	5 (17.9)	6 (17.6)	7 (12.3)	.7
CHA_2_DS_2_‐VASc score	2.46 (1.0, 3.0)	3.29 (1.0, 3.0)	2.31 (1.0, 3.0)	.51
Follow up				
Hospital admission rates (30 mo before enrolling)	3.3 ± 1.4	3.3 ± 1.3	3.1 ± 1.2	.79
Hospital admission rates during the FU	2.1 ± 1.3	2.0 ± 1.4	1.5 ± 1.3	.06
Baseline LVEF (%)	42.7 ± 3.8	40.8 ± 5.0	42.2 ± 4.5	.89
LVEF at last FU (%)	53.1 ± 5.5	50.2 ± 5.5	56.0 ± 4.0	.02
LVEDd at baseline (mm)	59.2 ± 4.0	57.0 ± 3.6	58.3 ± 4.8	.63
LVEDd at last FU (mm)	55.8 ± 2.9	54.1 ± 3.4	55.1 ± 3.5	.67
LAD at baseline (mm)	42.3 ± 4.6	42.2 ± 4.3	41.7 ± 4.0	.51
LAD at last FU (mm)	39.9 ± 5.0	39.3 ± 4.3	38.9 ± 4.4	.33
NYHA functional class at baseline	2.57 ± 0.57	2.79 ± 0.64	2.63 ± 0.56	.89
NYHA functional class at last FU	1.71 ± 0.66	1.61 ± 0.65	1.35 ± 0.52	<.01
Procedure and outcome				
All pulmonary veins isolated, no. (%)	29 (100)	36 (100)	55 (100)	1
Additional left atrial linear ablation, no. (%)	10 (35.7)	15 (44.1)	20 (34.5)	.67
Total duration of radiofrequency ablation (min)	86.7 ± 24.7	89.1 ± 22.8	93.0 ± 34.2	.33
Total duration of fluoroscopy (min)	15.6 ± 7.3	14.1 ± 5.5	14.6 ± 6.98	.6
Total duration of procedure (min)	174.6 ± 51.6	173.5 ± 39.70	176.9 ± 43.2	.78
Serious complications, N (%)				
Tamponade	2 (7.1)	1 (3.0)	1 (1.8)	.26
Stroke	0 (0.0)	0 (0.0)	0 (0.0)	1
Maintenance of sinus rhythm, N (%)	8 (10.3)	14 (40.0)	32 (58.2)	<.05

Abbreviations: AF, atrial fibrillation; CHA_2_DS_2_‐VASc, congestive heart failure, hypertension, age ≥75 (doubled), diabetes, stroke (doubled), vascular disease, age 65–74, and sex category (female); DCM, dilated cardiomyopathy; FU, follow up; LV, left ventricular; LVEDd, LV end of diastolic dimension; LVEF, LV ejection fraction; NYHA, New York Heart Association; SHD, structural heart disease; sTM, tachycardia‐related cardiomyopathy.

**Figure 3:**
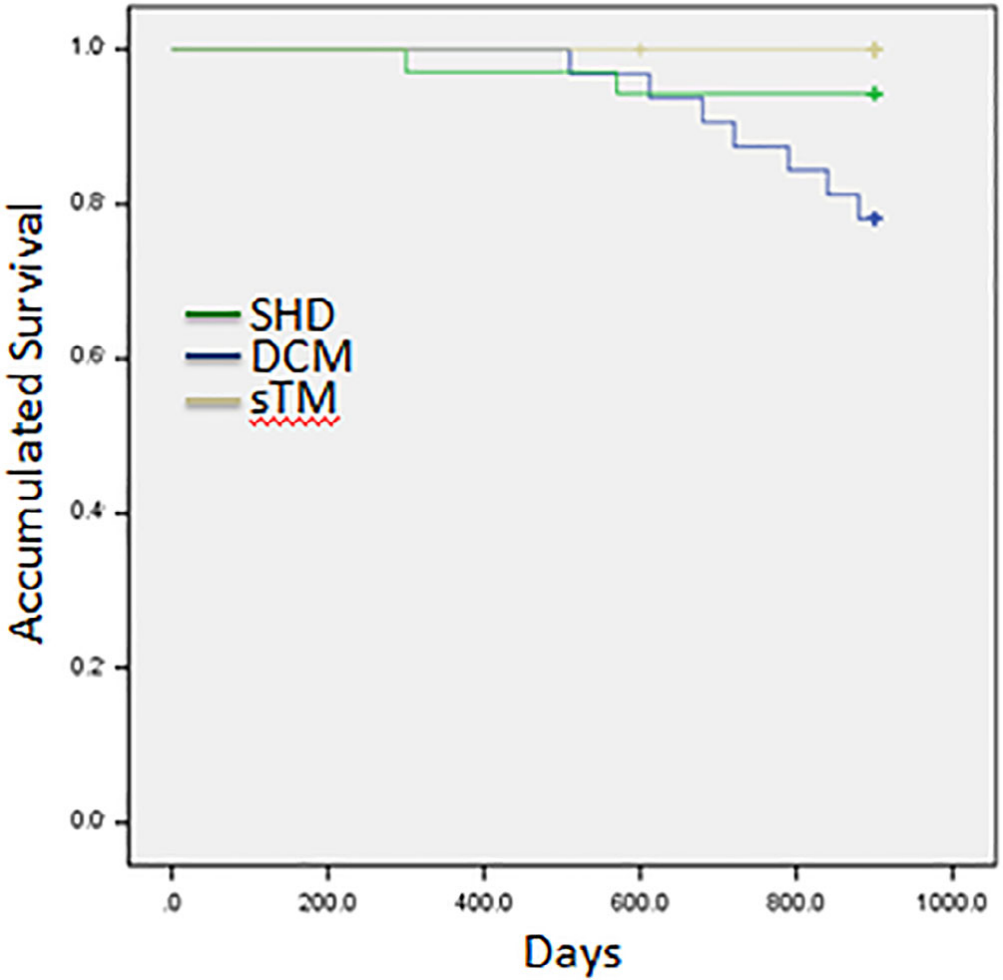
Accumulated survival curve among structural heart disease, dilated cardiomyopathy, and tachycardia‐related cardiomyopathy

After CA, the percentage of patients maintaining SR was significantly different among the three groups: 10.3% in DCM, 40.0% in SHD, and 58.2% in sTM (*P* < .01). Moreover, sTM had the highest improvement in LVEF, the NYHA class, and the percentage of patients maintained in SR among all heart diseases, and SHD had the least (*P* < .05 between changes). Regarding mortality, death occurred in five DCM, three SHD, and one sTM patients. Survival analysis via Kaplan‐Meier test estimated that DCM patients had significantly higher mortality than sTM patients (*P* < .05) after CA. However, the mortality was comparable between DCM vs SHD (*P* = .56) and SHD vs sTM (*P* = .14) (Figure 3). Furthermore, there was no significant difference in procedure parameters and complications among the three groups during the follow‐up period.

### 3.7 Predictors of SR maintenance in HF patients with CA

Univariate and multivariate analyses for predictors of SR maintenance in HF patients with CA are summarized in Table S3. Duration of AF history (OR: 0.64, 95% CI: 0.42‐0.96, *P* < .05) and the left atrial diameter (OR: 0.74, 95% CI: 0.65‐0.85, *P* < .001) were two consistent multivariate predictors for SR maintenance over the follow‐up periods, while the classification of AF (OR: 0.97, 95% CI: 0.95‐0.98, *P* < .001) was for the early recurrence of AF.

### 3.8 HF patients with paroxysmal AF benefit more from CA than nonparoxysmal AF

Therefore, to further understand which types of AF in HF patients benefit from the CA procedure, further analysis was conducted in patients with AFHF‐CA, which indicated that patients with paroxysmal AF had significantly higher SR maintenance rates than those with nonparoxysmal AF in the follow up of 12 months (36/38, 95% vs 42/82, 51%, *P* < .001), 24 months (32/38, 84% vs 36/82, 44%, *P* < .001), and 30 months (27/38, 72% vs 29/82, 35%, *P* < .001). The improvements in heart function were also significant in paroxysmal AF compared to nonparoxysmal AF (Figure S2), including NYHA functional class (*P* < .01), LVEF (*P* < .01), and LAD (*P* < .001), except LVEDd (*P* = .42). There was no significant difference in rehospitalization rate between paroxysmal and nonparoxysmal AF. All nine deaths in HFAF‐CA were recorded with nonparoxysmal AF (*P* < .05).

## 4 DISCUSSION

### 4.1 Main findings

In the present study, we retrospectively analyzed the efficacy of CA for AF in HF patients by comparing to those undergoing medical therapies and the safety of CA by comparing to those with normal heart function. The results indicated that (a) AFHF‐CA patients had a significantly higher rate of long‐term SR maintenance than those AFHF‐Med patients; (b) heart function improved significantly after CA in the AFHF‐CA group; (c) patients with CA had lower mortality and stroke incidence than those on medicine; and (d) CA in HF patients has comparable safety as CA in those patients with normal heart function, regarding CA‐related complications. Furthermore, subgroup analyses indicated that sTM patients might achieve the best benefit from CA, but the DCM patients had the least benefit. Moreover, in the AFHF‐CA group, paroxysmal AF had better prognosis and improvements in heart function than those with permanent or persistent AF.

### 4.2 Efficacy of CA in SR maintenance

Studies had shown that CA in AF patients with HF (AFHF‐CA) achieved significantly higher maintenance of SR. Previously, a randomized study in a small group of AF patients with HF achieved 81% freedom from AF at 6 months.^20^ A meta‐analysis from 26 studies reported that the success rate of CA was about 40% after a single procedure and up to 60% after multiple procedures in patients with LVEF <35% at a median 23‐month follow up.^21^ Most recently, a multicenter randomized study showed that CA in AF patient with HF (70%) is superior to amiodarone (34%) in achieving freedom from AF at the 24‐month follow up.^9^ In our study, we followed up the patients for 30 ± 6 months, and HF patients with CA had a significantly higher SR maintenance rate than those patients on medicine. Multiple factors, variables, and confounders might contribute to the SR maintenance, such as variations in study population with age; gender; AF types; LVEF value; etiology of HF; duration of AF and HF; and heterogeneous ablation strategies, procedures, and operator experience, as well as the difference in selection of ablation strategies, but CA showed independent benefits in AFHF patients.^9^, ^14^, ^20^, ^21^, ^22^ Bunch et al reported that 60.7% of patients had a clinical recurrence of AF after CA at 5‐year follow up.^14^ In the present study, we observed a similar SR maintenance rate after CA in patients with or without HF as previously reported (>50% vs >90%),^23^ and the progressive decreases along with time correlated with the duration of AF history, left atrial size, and types of AF. Therefore, the restoration and maintenance of SR should be the desired outcome for patients with AF and HF to achieve better prognosis. Mainly, AFHF‐Med had the highest risk of stroke among the three groups. Except for the lowest SR maintenance, the higher prevalence of hypertension, higher CHA_2_DS_2_‐Vasc‐score, and the higher rate of DCM might also contribute the risk of stroke in AFHF‐Med.^24^

### 4.3 Improvement of cardiac function by CA

Multiple studies showed significant improvements in cardiac function, HF hospitalizations, mortality, and stroke incidence.^14^, ^25^ A pooled absolute improvement in the LVEF from eight studies was 11% after CA.^25^ However, the mechanisms were controversial among studies. The maintenance of SR is essential for the improvement in the LVEF^14^ because the irregular heart rate itself may also lead to reduced cardiac output. Whether the type of AF contributes to the severity of reduced LVEF remained less clear, but the impaired LV systolic function could be improved after successful AF ablation and with SR.^19^ In our study, LVEF was significantly improved after a median follow up of 30 months in patients with SR. LVEF improvement appeared to be substantial in patients with SR. The low SR maintenance in patients on medical therapy during the follow up might mainly contribute to the worse prognosis of these patients due to the experience of AF recurrence and adverse events requiring the discontinuation of the drug as the previous trial suggested.^26^

### 4.4 Safety of CA in HF patients

Compared to patients with normal heart function, CA in patients with cardiac dysfunction required more additional ablation sites, which potentially increased the risk of the procedure‐related adverse events. The procedural complication ranged from 4.9% to 15.4%.^27^, ^28^, ^29^ In the present study, the rate of cardiac tamponade was comparable in patients with or without HF, 3.3% vs 2.7%. Moreover, no procedure‐related stroke and death were detected in patients with CA, which was comparable to the previous studies.^4^, ^20^

### 4.5 Benefits of CA in subgroups of patients

In previous studies, there is a high incidence of sTM associated with AF.^30^, ^31^ In the present study, subgroup analysis indicated that tachycardia‐related dysfunction benefited the most among the other etiologies, including significantly higher SR maintenance rates, lower mortality, and better improvements in cardiac function. Therefore, successful treatment by CA is recommended for patients with sTM and AF. Classification of AF, which is based on the duration of episodes and the burden of episodes, relies on diverse mechanisms. As in normal heart function, CA in HF showed a higher success rate and better prognosis in paroxysmal AF than in nonparoxysmal AF, in patients with short history of AF than long history, and in small left atria than in large atria, suggesting that consideration should be given to the selection of the strategies for AF therapy in HF patients individually. Pragmatic trial design for the precision AF therapy is warranted.^32^

### 4.6 Limitations

Our study is limited by the relatively small sample, limited follow‐up periods, and the retrospective design. To minimize bias, two groups of patients who were matched for age, gender, and type of AF were investigated as controls. The history of HF was not available for patients, which may affect the measurements in the improved cardiac function. Although ambulatory monitoring was used for the assessment of AF recurrence, it can never cover all recurrent AF, which may lead to an overestimated success rate. Repeated measurement on endpoints made up the limitations partially. This single‐center study was conducted retrospectively, and nonrandomized trials may not provide strong evidence as multiple randomized, controlled trials, but a real‐world study would provide pieces of evidence and answers to real‐world clinical practices.

## 5 CONCLUSIONS

CA in patients with HF had higher SR maintenance rates and reduced hospitalization rates, reduced mortality, and stroke events, as well as improvements in heart function, compared to medical therapies and had similar safety as inpatient without HF. Furthermore, patients with tachycardia‐related HF and paroxysmal AF had better clinical outcomes with CA strategy.

## 6 PERSPECTIVES

### 6.1 Competency in medical knowledge

Benefit of CA for AF is associated with lower mortality and higher survival rate free of AF. In those with HF, however, the benefit of CA for AF was a debate.

### 6.2 Translational outlook

Further randomized trials with stratified populations are needed to understand the differential outcome of CA in AF and to identify a subset of populations who would benefit from CA in safety and efficacy.

## CONFLICT OF INTEREST

The authors declare no potential conflict of interests.

## Supporting information


**Figure S1.** Kaplan‐Meier graph of maintenance of sinus rhythm during the follow up.

**Figure S2.** Improvements of heart function between paroxysmal and nonparoxysmal atrial fibrillation in heart failure patient with catheter ablation (AFHF‐CA) during the follow up.**P < .01(paroxysmal AF VS paroxysmal AF;nonparoxysmal AF VS nonparoxysmal AF ); ***P < .01(paroxysmal AF VS nonparoxysmal AF)

**Table S1.** Maintenance of sinus rhythm up to 30‐month follow up.
**Table S2.** Procedures and complications.
**Table S3.** Univariate and multivariate regression analyses of variables for predicting sinus rhythm maintenance at 12‐, 24‐, and 30‐month follow up in AHHF‐CA.
