# Supplementary figures and images for: Safety and efficacy of catheter ablation in atrial fibrillation patients with left ventricular dysfunction
Source: Clin Cardiol. 2019 Dec 5;43(3):305–14. doi: 10.1002/clc.23314 (PMC7068063; doi:10.1002/clc.23314)

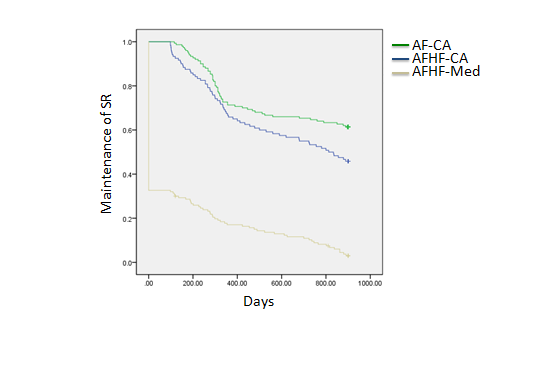

Supplement: Supplementary file 1 — Figure S1. Kaplan‐Meier graph of maintenance of sinus rhythm during the follow up. [file CLC-43-305-s001.png]

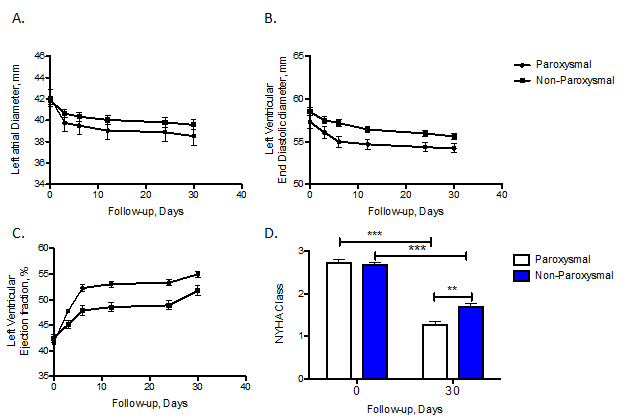

Supplement: Supplementary file 2 — Figure S2. Improvements of heart function between paroxysmal and nonparoxysmal atrial fibrillation in heart failure patient with catheter ablation (AFHF‐CA) during the follow up.**P < .01(paroxysmal AF VS paroxysmal AF;nonparoxysmal AF VS nonparoxysmal AF ); ***P < .01(paroxysmal AF VS nonparoxysmal AF) [file CLC-43-305-s002.png]
