# Supplementary material for: Safety and efficacy of catheter ablation in atrial fibrillation patients with left ventricular dysfunction
Source: Clin Cardiol. 2019 Dec 5;43(3):305–14. doi: 10.1002/clc.23314 (PMC7068063; doi:10.1002/clc.23314)
Supplement: Supplementary file 3 — Table S1. Maintenance of sinus rhythm up to 30‐month follow up. Table S2. Procedures and complications. Table S3. Univariate and multivariate regression analyses of variables for predicting sinus rhythm maintenance at 12‐, 24‐, and 30‐month follow up in AHHF‐CA. [file CLC-43-305-s003.docx]

**Supplemental Table I**  Maintenance of sinus rhythm (SR) during up to 30-month follow-up

| Items | AF-CA(150) | AFHF-CA(120) | AFHF-Med(150) | P Value | P Value |
| --- | --- | --- | --- | --- | --- |
| Maintenance of sinus rhythm(SR),N(%) |  |  |  |  |  |
| The SR in 12 months,N(%) | 107(71) | 79(66) | 25(17) | 0.27 | ＜0.01 |
| The SR in 24 months,N(%) | 98(65) | 68(57) | 16(11) | 0.15 | ＜0.01 |
| The SR in 30months,N(%) | 92(61) | 56(47) | 4(27) | ＜0.05 | ＜0.01 |

**Supplemental Table II**  Procedures and complications

| **Items** | AF-CA | AFHF-CA | P Value |
| --- | --- | --- | --- |
| Pulmonary veins isolated,N (%) | 150（100） | 120（100） | 1 |
| Additional left atrial linear ablation ,N (%) | 107（71） | 103（86） | ＜0.01 |
| No extra line | 43(29) | 17(14) | 0.23 |
| 1 | 46(31) | 54(45) | 0.15 |
| 2 | 1(0.7) | 0(0) | - |
| 3 | 59(39) | 48(40) | 0.92 |
| 4 | 1(0.7) | 1(0.8) | - |
| Total duration of radiofrequency ablation ,min | 83.6±21.2 | 89.8±28.8 | ＜0.05 |
| Total duration of fluoroscopy, min | 14.7±9.6 | 14.6±6.6 | 0.9 |
| Total duration of procedure ,min | 166.8±36.7 | 175.2±43.8 | 0.09 |
| Serious complications,N (%) |  |  |  |
| Tamponade | 4（2.6） | 4（3.2） | 0.51 |
| Stroke | 0（0） | 0（0） | - |

**Supplemental Table III**: Univariate and multivariate regression analysis of variables for predicting sinus rhythm maintenance at 12-, 24-, and 30- months follow-up in AHHF-CA.

| Variables | 12-month follow-up | | | | | 24-month follow-up | | | | | | 30-month follow-up | | | | | | |
| --- | --- | --- | --- | --- | --- | --- | --- | --- | --- | --- | --- | --- | --- | --- | --- | --- | --- | --- |
|  | Univariate analysis | | | Multivariate analysis | | Univariate analysis | | Multivariate analysis | | | Univariate analysis | | | | Multivariate analysis | | |  |
|  | P value | Odds Ratio | P value | | Odds Ratio | P value | Odds Ratio | P value | Odds Ratio | P value | | | Odds Ratio | P value | | Odds Ratio |  |  |
| Age, year | <0.001 | 0.89 (0.84-0.95) | 0.95 | | 1.0 (0.90-1.12) | <0.001 | 0.88 (0.83-0.93) | 0.85 | 1.01 (0.90-1.13) | 0.001 | | | 0.92 (0.87-0.96) | 0.88 | | 1.0 (0.9-1.13) |  |  |
| Male | 0.76 | 0.88 (0.41-1.91) |  | |  | 0.41 | 1.36 (0.65-2.85) |  |  | 0.3 | | | 1.48 (0.71-3.09) |  | |  |  |  |
| Classification of atrial fibrillation | 0.001 | 0.43 (0.26-0.70) |  | |  | 0.003 | 0.52 (0.33-0.80) | 0.07 | 0.44 (0.19-1.05) | 0.03 | | | 0.64 (0.42-0.96) | 0.13 | | 0.54 (0.24-1.2) |  |  |
| Duration of atrial fibrillation, month | <0.001 | 0.97 (0.96-0.98) | <0.001 | | 0.97 (0.96-0.99) | <0.001 | 0.96 (0.96-0.97) | <0.001 | 0.97 (0.95-0.98) | <0.001 | | | 0.97 (0.95-0.98) | <0.001 | | 0.97 (0.96-0.98) |  |  |
| Electrical cardioversion, Times | 0.07 | 0.47 (0.21-1.06) |  | |  | 0.08 | 0.49 (0.22-1.09) |  |  | 0.4 | | | 0.71 (0.32-1.57) |  | |  |  |  |
| Antiarrhythmic drugs tried, Times | 0.003 | 0.1 (0.02-0.45) | 0.39 | | 0.43 (0.06-2.98) | 0.001 | 0.15 (0.05-0.48) | 0.37 | 0.40 (0.06-2.89) | 0.002 | | | 0.21 (0.08-0.56) | 0.35 | | 0.39 (0.06-2.75) |  |  |
| Treatment with amiodarone | 0.15 | 4.69 (0.57-38.8) |  | |  | 0.2 | 2.87 (0.57-14.4) |  |  | 0.03 | | | 10.1 (1.22-83.6) |  | |  |  |  |
| CHA 2 DS 2 -Vasc-score | 0.006 | 0.59 (0.40-0.86) | 0.26 | | 1.62 (0.70-3.72) | <0.001 | 0.46 (0.31-0.7) | 0.26 | 1.61 (0.7-3.7) | 0.002 | | | 0.54 (0.36-0.8) | 0.63 | | 8.7 (0.01-6.8) |  |  |
| Cardiovascular diseases, (number) | 0.04 | 0.59 (0.35-0.98) | 0.07 | | 0.38 (0.13-1.08) | 0.02 | 0.54 (0.33-0.89) | 0.06 | 0.37 (0.13-1.04) | 0.01 | | | 0.52 (0.31-0.86) | 0.09 | | 0.42 (0.15-1.14) |  |  |
| Hypertension | 0.76 | 0.88 (0.41-1.91) |  | |  | 0.5 | 0.77 (0.37-1.63) |  |  | 0.65 | | | 0.85 (0.41-1.75) |  | |  |  |  |
| Diabetes mellitus | 0.09 | 0.42 (0.15-1.13) |  | |  | 0.06 | 0.38 (0.14-1.05) |  |  | 0.05 | | | 0.34 (0.11-1.0) |  | |  |  |  |
| Dilated cardiomyopathy | 0.21 | 0.58 (0.25-1.35) |  | |  | 0.54 | 0.76 (0.33-1.78) |  |  | 0.24 | | | 0.60 (0.25-1.41) |  | |  |  |  |
| Coronary artery disease | 0.82 | 0.88 (0.30-2.62) |  | |  | 0.27 | 0.55 (0.19-1.59) |  |  | 0.39 | | | 0.62 (0.21-1.84) |  | |  |  |  |
| Valvular disease | 0.89 | 1.08 (0.35-3.42) |  | |  | 0.78 | 0.86 (0.29-2.54) |  |  | 0.95 | | | 0.96 (0.33-2.84) |  | |  |  |  |
| Congenital heart disease | 0.66 | 0.53 (0.03-8.74) |  | |  | 0.85 | 0.76 (0.05-12.5) |  |  | 0.71 | | | 0.93 (0.65-1.34) |  | |  |  |  |
| Hypertrophic cardiomyopathy | 0.28 | 0.26 (0.02-2.95) |  | |  | 0.43 | 0.37 (0.03-4.23) |  |  | 0.62 | | | 0.54 (0.05-6.17) |  | |  |  |  |
| Heart Function |  |  |  | |  |  |  |  |  |  | | |  |  | |  |  |  |
| NYHA functional class | 0.45 | 0.78 (0.40-1.49) |  | |  | 0.64 | 0.86 (0.46-1.61) |  |  | 0.76 | | | 0.91 (0.49-1.69) |  | |  |  |  |
| Left ventricular ejection fraction, % | 0.1 | 1.07 (0.99-1.16) |  | |  | 0.17 | 1.05 (0.98-1.15) |  |  | 0.23 | | | 1.06 (0.97-1.14) |  | |  |  |  |
| End-diastolic Left ventricular dimensions, mm | 0.02 | 0.90 (0.82-0.98) | 0.58 | | 0.95 (0.81-1.13) | 0.03 | 0.91 (0.83-0.99) | 0.65 | 0.96 (0.82-1.13) | 0.11 | | | 0.93 (0.85-1.01) | 0.09 | | 0.42 (0.15-1.14) |  |  |
| Left atrial parasternal dimension, mm | 0.002 | 0.86 (0.77-0.95) | 0.01 | | 0.84 (0.72-0.97) | <0.001 | 0.79 (0.71-0.89) | 0.02 | 0.84 (0.72-0.97) | <0.001 | | | 0.74 (0.65-0.85) | 0.02 | | 0.85 (0.74-0.97) |  |  |
| Additional line ablation | 0.91 | 1.02 (0.74-1.41) |  | |  | 0.81 | 1.04 (0.76-1.42) |  |  | 0.52 | | | 1.10 (0.821-1.51) |  | |  |  |  |
| Repeated ablation | 0.45 | 0.62 (0.18-2.15) |  | |  | 0.88 | 0.91 (0.26-3.16) |  |  | 0.89 | | | 0.91 (0.26-3.17) |  | |  |  |  |
